# Development of nanosecond spike pulse power supply for electrochemical micromachining

**DOI:** 10.1038/s41598-023-48793-z

**Published:** 2023-12-21

**Authors:** Chuanjun Zhao, Qingzhi Zou, Xiaoguang Ren, Lizhong Xu

**Affiliations:** 1 School of Mechanical Engineering, Yanshan University, Qinhuangdao, 066004 China; 2 College of Mechanical and Electrical Engineering, Hebei Normal University of Science & Technology, Qinhuangdao, 066004 China

**Keywords:** Engineering, Electrical and electronic engineering, Mechanical engineering

## Abstract

The use of pulse voltage can greatly improve the precision of electrochemical microfabrication, and the narrower the pulse width of the applied pulse voltage signal, the higher the machining precision. However, the commonly used chopper circuit topology of pulse power supplies is limited by the maximum switching frequency of the field-effect transistor. To address this problem, this paper proposes a nanosecond pulse electrochemical micromachining power supply based on a differential circuit. The power supply uses the STM32F103C8T6 microcontroller as the control core to output high-performance rectangular waves through a DDS device. After differential, rectification, filtering, and power amplification processing, stable, frequency, amplitude, and pulse width adjustable spike pulse voltage signals are obtained. By establishing a system mathematical model and optimizing the time constant of the differential circuit, theoretically, the sub-nanosecond pulse width can be obtained. Prototype performance tests show that the power supply has a maximum frequency of 20 MHz, a minimum pulse width of 1.8 ns, and a maximum peak voltage of 10 V. By using this power supply for microhole electrochemical machining experiments, nanometer-level machining precision has been achieved.

## Introduction

Electrochemical micromachining (ECMM) is a non-contact special machining technology based on the principle of electrochemical anode solution. It has the advantages of tool wear-free, residual stress-free, and not limited by material hardness. During the machining process, the anode is eroded in ionic form^1,2^. Theoretically, ECMM can achieve nanometer-level machining accuracy and is an ideal method for processing high-precision micro-parts, difficult-to-machine materials, and complex-shaped structures. It has broad application prospects in high-tech fields such as micro-robotics, MEMS, aerospace technology, and military industry^3–5^.

The power supply is a critical component of the electrochemical machining (ECM) system. Initially, direct current (DC) power was used in ECM, which had high shaping efficiency but limited machining accuracy of 0.2–0.7 mm. In 1967, Cook et al.^6^ from the University of Cambridge designed a Pulse Electrochemical Machining (PECM) system to remove the workpiece's passivation layer by using reverse voltage. The application of a pulse power supply further improved the precision of ECM. In the early days of PECM, intermittent low-frequency (tens of hertz) and wide-pulse (milliseconds) electric current was used to improve the machining accuracy to about 0.1 mm, but the machining efficiency was low, and the pulse effect was not fully demonstrated^7^. It was not until 2000 that Schuster et al. from Germany^8^ milled high-precision complex three-dimensional structures on copper sheets with a frequency of 2 MHz and a pulse width of 50 ns using ultra-short pulse voltage, with the ECM gap controlled at 2 μm. The application of high-frequency and ultra-short pulse power supplies has truly enabled ECM to enter the field of microfabrication.

When the electrolyte parameters are determined, the accuracy of PECM mainly depends on the pulse width of the applied voltage signal, with narrower pulse width resulting in higher machining accuracy. In theory, nanometer-scale machining precision can only be achieved with pulse widths in the picosecond range^9^. Currently, the main circuit topologies of ECMM ultra-short pulse power supplies include the chopper type and power amplifier type. The chopper type is to convert the stable DC voltage signal into a pulse voltage signal with the same switching frequency through the on–off action of the high-frequency switching device.

However, due to the technical limitation of the maximum switching frequency of the metal–oxide–semiconductor field-effect transistor (MOSFET) in the chopper circuit^10^ and the influence of stray inductance and capacitance^11^, the pulse width cannot be shortened without limit, which limits the accuracy of ultrashort PECMM further improvement. The power amplifier type directly amplifies the ultra-short pulse signal generated by the pulse generator to obtain the ultra-short pulse voltage required for PECM^12^. This scheme has a relatively simple main circuit, but lacks an interface for the control system, resulting in poor performance in terms of short-circuit protection response time and expandability.

Currently, ultra-short pulse power sources are still in the development stage. Chang et al.^13^ developed a nanosecond pulse power source with a minimum pulse width of 50 ns, using the STM32F103VET6 single-chip computer as the control core and metal–oxide–semiconductor field-effect transistors as the pulse cutting switch components. Mole et al.^14^ developed a nanosecond pulse power source that, through closed-loop control of current and voltage, is capable of generating pulse voltages with a maximum pulse width of 45 MHz and a minimum of 14 ns. Giandomenic et al.^15^ utilized a fixed frequency buck converter (LT3590) to provide stable DC voltage and generated pulse voltage with a pulse width of 10–500 ns through MOSFET chopping. Zhang et al.^16^ developed a high-frequency pulse power supply by using the precision function waveform generator MAX038 and a high-speed MOSFET chopper complementary. The power supply voltage range is 1.5–10 V, and the minimum output pulse width is 50 ns. Li et al.^17^ proposed and designed a Positive and Negative Pulse (PANP) power source, which enhances micro-hole machining precision and reduces overcut through the negative pulse effect.

As the pulse width decreases, the influence of external circuit parameters such as stray capacitance and inductance on the pulse characteristics becomes increasingly prominent. When the pulse width decreases to the order of nanoseconds, the rise time and fall time of the pulse signal is significantly prolonged due to the influence of stray capacitance and inductance, causing pulse waveform distortion^16,18^. Therefore, it is difficult to obtain an ideal pulse signal with a pulse width of nanoseconds or sub-nanoseconds using conventional main circuit topologies.

Based on the analysis of the electrochemical micromachining mechanism, this paper proposes a nanosecond spike pulse circuit topology for ECMM. By using differential circuits to process the rectangular pulse signal and optimizing its parameters, the spike pulse voltage width can be shortened to 1 ns or even sub-nanosecond level. The topology of the spike pulse power supply circuit is simple, cost-effective, and stable, which can further improve the precision and localization of PECMM.

## Overall structure design

**Figure 1:**
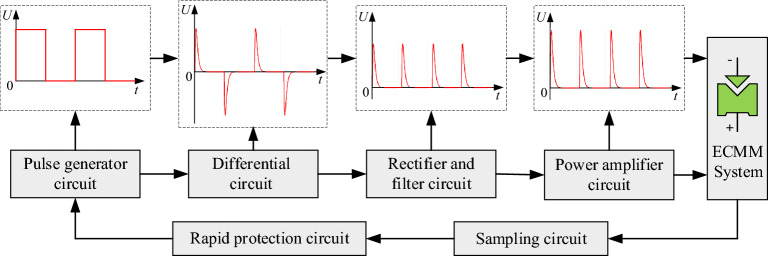
Schematic diagram of spike power supply.

The principle of the spike pulse power supply is shown in Fig. 1, which mainly includes the pulse generating circuit, differentiation circuit, rectification circuit, and power amplification circuit. The pulse generating circuit generates rectangular pulse voltage signals with adjustable frequency, amplitude, and duty cycle through a microcontroller and a DDS module. After being processed by the differentiation circuit, the rectangular pulse signal is converted into high-frequency and narrow-pulse-width AC spike pulse voltage signals. Then, the rectification circuit filters out the negative voltage to obtain positive spike pulse signals that are consistent with the input pulse signal frequency. Finally, the obtained positive spike pulse is amplified by the power amplification circuit to obtain a high-frequency spike pulse voltage signal that can be directly used for ECMM. In addition, the overall structure of the spike pulse power supply also includes a detection circuit and a rapid protection circuit. When a short circuit occurs in the ECMM, the protection circuit quickly controls the power outage, which is used to protect the pulse power supply, cathode electrodes, and anode workpieces.

### Pulse generation circuit design

**Figure 2:**
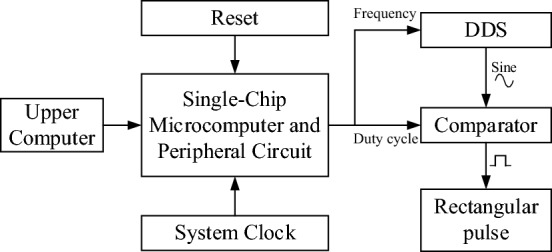
Schematic diagram of pulse generation circuit.

Figure 2 depicts the principle diagram of the pulse generator circuit. The single-chip microcomputer communicates with the Direct Digital Synthesizer (DDS) module via the Serial Peripheral Interface (SPI), which enables the microcontroller to control the generation of a sinusoidal signal with a specific frequency and amplitude. Subsequently, a comparator module generates a rectangular pulse signal with an adjustable duty cycle. The single-chip microcomputer outputs a rectangular pulse signal with adjustable frequency, amplitude, and duty cycle through the serial port communication with the upper computer.

**Figure 3:**
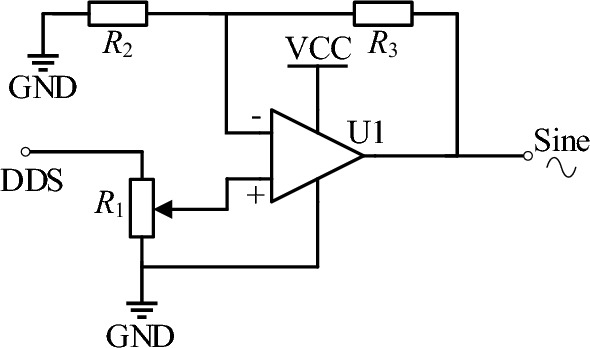
Sine signal generating circuit.

The single-chip microcomputer selects STM32F103C8T6 designed by STMicroelectronics, which is a 32-bit microcontroller based on the ARM Cortex-M3 core. The DDS module utilizes the low-power device AD9851 from Analog Devices Inc. The AD9851’s innovative high-speed DDS core accepts a 32-bit frequency tuning word, which results in an output tuning resolution of approximately 0.04 Hz with a 180 MHz system clock. At its highest clock frequency, the device consumes only 555mW of power, with rise and fall times as short as 3.5 ns. The circuit for generating a sine wave is shown in Fig. 3. According to actual tests, the amplitude of the output sine wave is approximately 0.12 V at 20 MHz. To achieve a fixed-frequency waveform output, a gain-adjustable operational amplifier module is added to the system. The digital potentiometer is controlled by the microcontroller to adjust the amplitude of the Sine signal, thus enabling a constant output of a 1 Vpp sine wave with a maximum frequency of 20 MHz.

**Figure 4:**
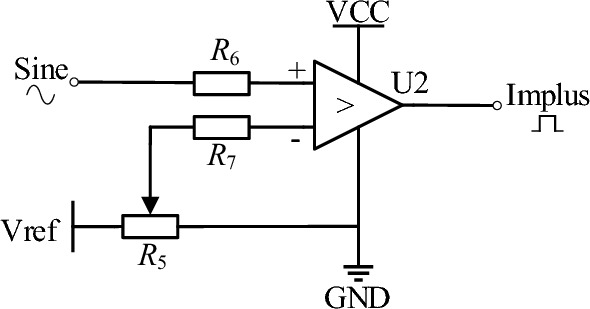
Comparator circuit.

The comparator circuit is shown in Fig. 4. The sine wave signal outputted by DDS is fed into the high-speed comparator module, which outputs a rectangular pulse with an adjustable amplitude of 5 V and a duty cycle ranging from 0 to 50%. The amplitude of the input sinusoidal wave is fixed at 0.5 V, while Vref is the reference voltage of the comparator, with a value of 0.5 V. The duty cycle of the output pulse signal can be adjusted by the digital potentiometer *R*_5_. When an 8-bit digital potentiometer MCP41010 is used for *R*_5_, the duty cycle adjustment accuracy is 0.0039%.

### Differential circuit design

The differential circuit is the core component that converts rectangular pulse signals into spike pulse waveforms. A differential circuit can significantly reduce pulse width because it only generates an output at the moment when the input waveform experiences a sudden change, while no output is produced during the steady state. The width of the spike pulse waveform output by the differential circuit depends on its time constant *τ* (*τ* = *RC*). The smaller the time constant, the narrower the spike pulse waveform, and vice versa. To achieve waveform transformation, the time constant of the differential circuit should be smaller than the duration of the rectangular pulse waveform *t*_on_. Generally, a time constant *τ* that is less than or equal to 1/10 of the input waveform duration is selected.

#### Differential circuit model

**Figure 5:**
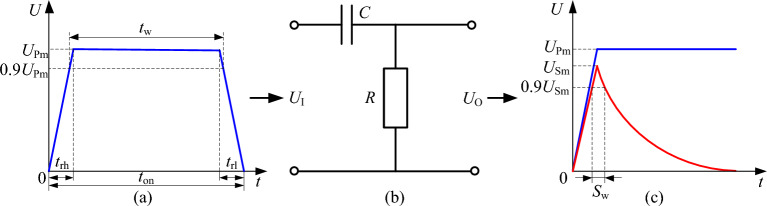
Differential circuit characteristics. (**a**) Input rectangular pulse waveform; (**b**) Differential circuit; (**c**) Output spike waveform.

The principle of waveform transformation in differential circuits is shown in Fig. 5. Let *U*_Pm_ be the peak voltage of the input rectangular pulse signal, *t*_rh_ and *t*_rl_ be the rise time and fall time, respectively, *t*_on_ be the pulse duration, and *t*_w_ be the pulse width (the duration of the pulse voltage reaching 90% of the peak voltage), as shown in Fig. 5a.

According to electrical knowledge, the step response of differential circuits is (The detailed derivation process can be found in the supplementary material):

1 $$U_{{\text{S}}} (t) = \left\{ \begin{aligned} & \frac{{U_{{{\text{Pm}}}} \tau }}{{t_{{{\text{rh}}}} }}\left( {1 - e^{{ - \frac{t}{\tau }}} } \right){, 0 < }t \le t_{{{\text{rh}}}} \hfill \\ & U_{{{\text{Sm2}}}} e^{{ - \frac{{t - t_{{{\text{rh}}}} }}{\tau }}} {, }t_{{{\text{rh}}}} { < }t \le t_{{{\text{on}}}} - t_{{{\text{rl}}}} \hfill \\ & - \frac{{U_{{{\text{Pm}}}} }}{{t_{{{\text{rl}}}} }}\left( {1 - e^{{ - \frac{{t{ - }t_{{{\text{on}}}} + t_{{{\text{rl}}}} }}{\tau }}} } \right){ + }U_{{{\text{Sm3}}}} {, }t_{{{\text{on}}}} - t_{{{\text{rl}}}} { < }t \le t_{{{\text{on}}}} \hfill \\ & U_{{{\text{Sm4}}}} e^{{ - \frac{t}{\tau }}} , \, t_{{{\text{on}}}} { < }t \le T \hfill \\ \end{aligned} \right.$$

When *t* = *t*_rh_, *t* = *t*_on_-*t*_rl_, and *t* = *t*_on_, the spike pulse output voltage reaches extreme values of *U*_Sm2_, *U*_Sm3_, and *U*_Sm4_, respectively, which are calculated as follows:

2 $$\left\{ \begin{aligned} & U_{{{\text{Sm2}}}} = \frac{{U_{{{\text{Pm}}}} \tau }}{{t_{{{\text{rh}}}} }}\left( {1 - e^{{ - \frac{{t_{{{\text{rh}}}} }}{\tau }}} } \right) \hfill \\ & U_{{{\text{Sm3}}}} = U_{{{\text{Sm2}}}} e^{{ - \frac{{t_{{{\text{on}}}} - t_{{{\text{rl}}}} - t_{{{\text{rh}}}} }}{\tau }}} \hfill \\ & U_{{{\text{Sm4}}}} = - \frac{{U_{{{\text{Pm}}}} }}{{t_{{{\text{rl}}}} }}\left( {1 - e^{{ - \frac{{t_{{{\text{rl}}}} }}{\tau }}} } \right){ + }U_{{{\text{Sm3}}}} \hfill \\ \end{aligned} \right.$$

The duration of the spike pulse exceeding 90% of the peak voltage, defined as the pulse width *S*_w_, is shown in Fig. 5c. The pulse width of the spike pulse can be obtained as:

3 $$S_{{\text{w}}} = \tau \ln \left( {0.1 + 0.9e^{{ - \frac{{t_{{{\text{rh}}}} }}{\tau }}} } \right) - \tau \ln (0.9) + t_{{{\text{rh}}}}$$

The main design parameters of the spike voltage signal include frequency, amplitude, and pulse width. The frequency of the spike voltage signal depends on the frequency of the rectangular wave input to the differential circuit and can be adjusted by the pulse generator circuit. Equation (2) shows that the pulse width of the spike pulse *S*_w_ is only related to the rise time *t*_rh_ of the rectangular pulse, and the time constant *τ*. Since it is difficult to adjust the rise time of the rectangular pulse *t*_rh_, the time constant of the differential circuit *τ* is the key parameter that needs to be optimized in the design process of the spike pulse power supply topology.

#### Time constant optimization

**Figure 6:**
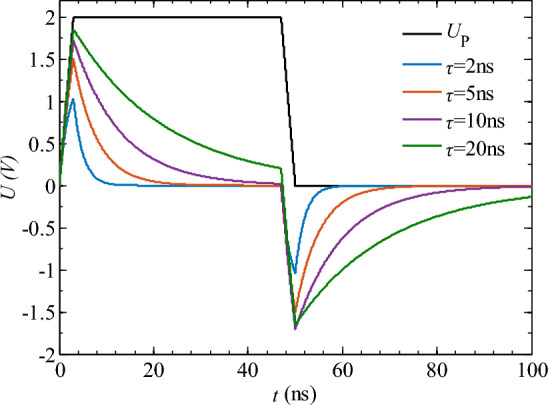
Output response characteristics of differential circuits. *U*_P_ is the input rectangular pulse voltage waveform.

Assuming a rectangular pulse with a peak voltage of *U*_Pm_ = 2 V, rise/fall times of *t*_rh_ = *t*_rl_ = 3.5 ns, period *T* = 100 ns, and pulse duration *t*_on_ = 50 ns, the waveform of *U*_P_ is obtained using Eq. (1) as shown in Fig. 6. Based on the output response function of the differential circuit (Eqs. (1) and (2)), we take differential circuit time constants of *τ* = 2, 5, 10, and 20 ns to investigate the output response of the spike pulse power supply, as shown in Fig. 6. Since there is a reverse output response in the differential circuit during the intermittent stage of the rectangular pulse, the duty cycle of the input rectangular pulse signal is set to 50%.

**Figure 7:**
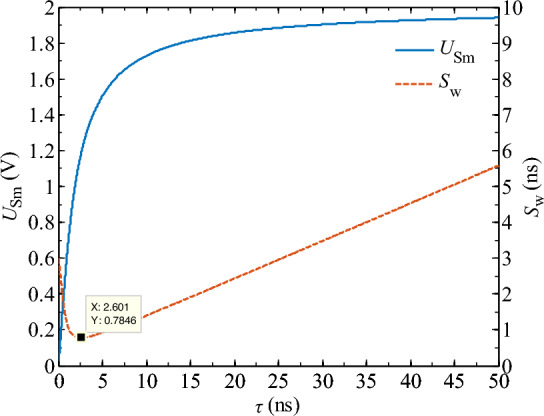
The variation of spike pulse width *S*_on_ and peak voltage *U*_Sm_ with time constant *τ.*

Figure 6 shows that as the time constant *τ* decreases, the peak voltage response of the spike pulse *U*_Sm_ decreases and the spike pulse width becomes narrower. When the time constant *τ* = 20 ns, the capacitor has not fully charged and enters the pulse-off phase. To complete the spike pulse waveform transformation properly, the time constant *τ* should be at least less than the duration of the input rectangular pulse *t*_on_, i.e., *τ* ≤ *t*_on_. According to Eqs. (3), the time constant *τ* determines the pulse width *S*_w_ and peak voltage *U*_Sm_ of the spike pulse. Let the peak voltage of the input rectangular pulse *U*_Pm_ = 2 V and the duration of the pulse *t*_on_ = 50 ns, with a rise time *t*_rh_ = 3.5 ns. Using Matlab, the variation of *S*_w_ and *U*_Sm_ with *τ* can be obtained as shown in Fig. 7.

**Figure 8:**
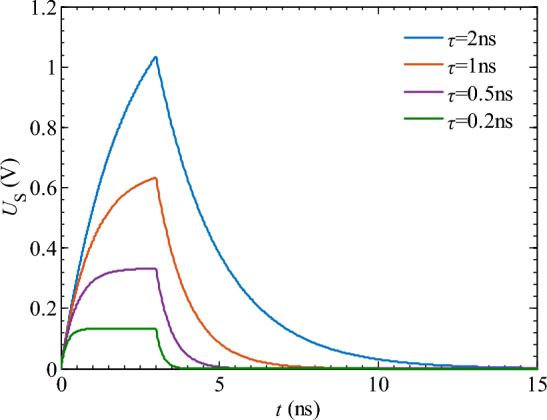
The voltage output waveform of the differential circuit when *τ* < 2.6 ns.

Figure 7 shows that as the time constant increases, the spike pulse width *S*_w_ first decreases sharply and then gradually increases. When the time constant *τ* = 2.6 ns, the spike pulse width *S*_w_ reaches its minimum value of 0.78 ns. This is because when the time constant *τ* is small, the response speed of the differentiator circuit is extremely fast, and the capacitor of the differentiator circuit can be charged during the rising stage of the rectangular pulse (*t* ≤ *t*_rh_). The output waveform of the differentiator circuit reaches a stable state, which is no longer a spike pulse, but rather a rectangular pulse with a small amplitude (as shown in Fig. 8). Therefore, during the 0 < *t* < 2.6 ns stage, the spike pulse width *S*_w_ decreases with an increase in the time constant *τ*. Based on the above analysis, to ensure the proper transformation of the spike pulse waveform, the time constant *τ* should be set to at least 2.6 ns.

To achieve higher machining accuracy and localization in PECMM, it is necessary to reduce the material removal per pulse of the anode by minimizing the pulse width of the machining power supply.To balance the contradiction between peak voltage *U*_Sm2_ and pulse width *S*_w_, the selection of the time constant *τ* of the differential circuit is optimized by introducing a multiplication and division optimization objective function *Y*. The definition of *Y* is as follows:

4 $$Y = \frac{{U_{{{\text{Sm2}}}} }}{{S_{{\text{w}}} }}{ = }\frac{{U_{{{\text{Pm}}}} \tau }}{{\tau t_{{{\text{rh}}}} \ln (0.1 + 0.9e^{{ - \frac{{t_{{{\text{rh}}}} }}{\tau }}} ) - \tau t_{{{\text{rh}}}} \ln (0.9) + t_{{{\text{rh}}}}^{2} }}\left( {1 - e^{{ - \frac{{t_{{{\text{rh}}}} }}{\tau }}} } \right)$$

**Figure 9:**
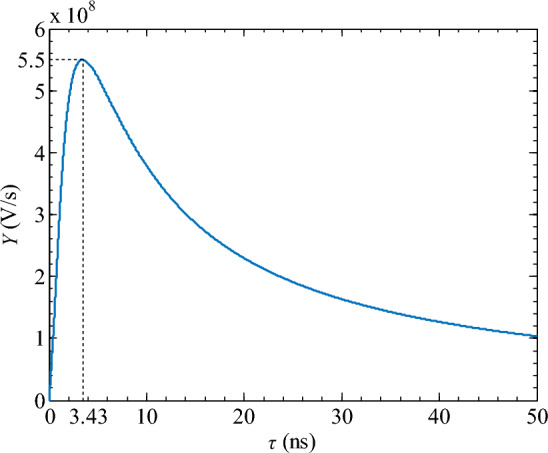
Functional relationship between objective function Y and time constant *τ*.

In Eq. (4), the unit of the objective function *Y* is V/s, which can be regarded as the rate of voltage change. When the objective function *Y* reaches its maximum value, it can ensure that the differential circuit obtains a narrow pulse width without excessive voltage drop. Taking *U*_Pm_ = 2 V and *t*_rh_ = 3.5 ns, the relationship between the objective function *Y* and the time constant *τ* can be obtained by solving Eq. (4), as shown in Fig. 9.

Figure 9 shows that the objective function *Y* is a unimodal curve. The maximum value of *Y*_max_ = 5.5 × 10^8^ V/s is obtained when the time constant *τ* = 3.43 ns. By substituting *τ* = 3.43 ns into Eqs. (2) and (3), the peak voltage *U*_Sm2_ and the spike pulse width *S*_w_ can be obtained:

5 $$\left\{ \begin{gathered} U_{{{\text{Sm2}}}} = 1.33{\text{V}} \hfill \\ S_{{\text{w}}} = 0.81{\text{ns}} \hfill \\ \end{gathered} \right.$$

**Figure 10:**
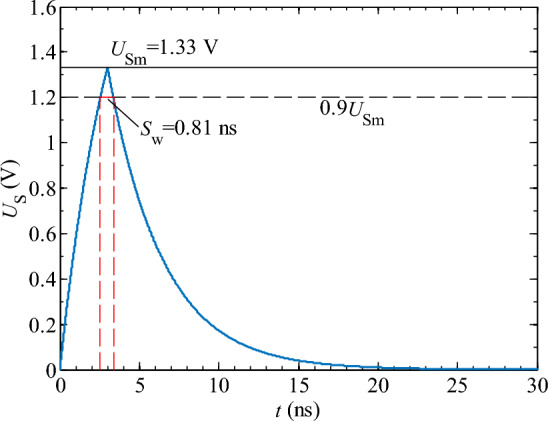
Optimized spike pulse waveform. (*τ* = 3.43 ns; *U*_Pm_ = 2 V; *t*_r_ = 3 ns; *t*_on_ = 50 ns).

Through optimization analysis of the objective function, it was found that by setting the time constant of the differential circuit to 3.43 ns, a rectangular pulse signal with a duration of 50 ns and peak voltage of 2 V can be converted to a narrow pulse signal with a pulse width of 0.81 ns and peak voltage of 1.33 V (as shown in Fig. 10). After a comprehensive analysis, the optimal range of time constant *τ* for the differential circuit is between 2.6 ns and 0.1 *t*_on_ (*t*_on_ ≥ 26 ns) to improve the precision of ECMM and ensure complete transformation of the spike pulse waveform.

### Rectification and filter circuit design

**Figure 11:**
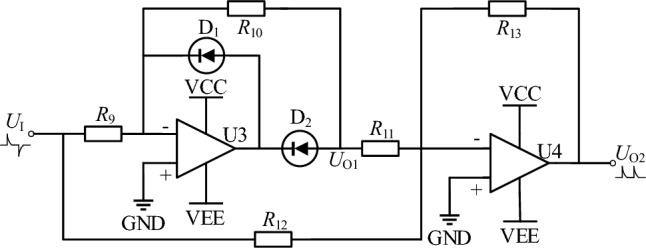
Precision full wave rectifier circuit.

The existence of differential circuits causes significant reverse spikes during the interval when rectangular pulse signals enter the off period. These negative pulses can corrode and damage the cathode electrode, which can affect the shape and size accuracy of the processed anode. To eliminate the reverse voltage generated by the differential circuit, a precision full-wave rectifier circuit is introduced, as shown in Fig. 11. In Fig. 11, D1 and D2 are super fast surface mount rectifiers (1N4148) with a maximum reverse recovery time of less than 4 ns. Integrated operational amplifiers U_3_ and U_4_ use OPA695 with a gain bandwidth of 1.7 GHz, a slew rate of 4300 V/μs, and a rise/fall time of 1 ns.

**Figure 12:**
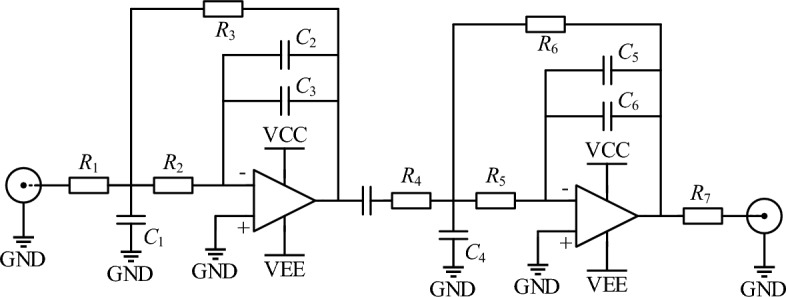
Precision full wave rectifier circuit.

To filter out high-frequency interference signals generated during signal processing, a fourth-order active Butterworth filter with a cut-off frequency of 30 MHz was selected. The main circuit of the filter is shown in Fig. 12. The filter uses a two-stage active filtering method, which results in a flat gain within the passband and a sharp cut-off at the cut-off frequency, resulting in good filtering performance.

To eliminate high-frequency interference signals generated during signal processing, a fourth-order active Butterworth filter with a cut-off frequency of 30 MHz was chosen. The main circuit of the filter is illustrated in Fig. 12. The filter uses a two-stage active filtering approach, which effectively eliminates unwanted signals and enhances the overall signal quality with its sharp cut-off and high attenuation.

### Power amplifier circuit design

**Figure 13:**
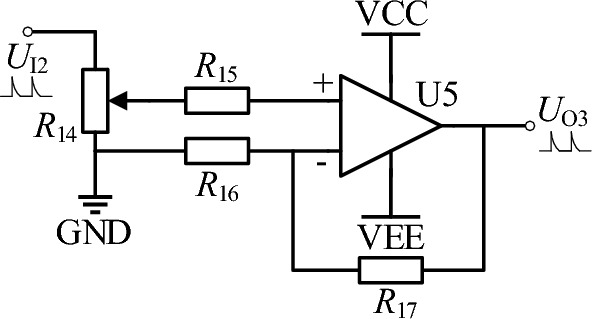
Power amplifier circuit.

The power of the rectified and filtered spike pulse voltage signal is small, and its load capacity is weak, which cannot meet the requirements of ECMM. Therefore, a power amplifier circuit is needed to amplify the spike pulse voltage and current signals. Figure 13 shows the schematic diagram of the power amplifier circuit, where *R*_14_ is an 8-bit digital potentiometer used to adjust the peak voltage of the output signal of the power amplifier circuit. The voltage gain is $A_{{\text{V}}} = 1 + \frac{{R_{17} }}{{R_{16} }}$. U_5_ uses a high-speed operational amplifier PA119, with a gain bandwidth of 350 MHz, a slew rate of 900 V/μs, and a peak current of up to 4A. The maximum output signal peak value of the power amplifier circuit is *U*_max_ = 3*A*_V_, which can be adjusted within this range with an adjustment accuracy of 0.06%.

### Fast short circuit protection circuit design

For higher precision in ECMM, the machining gap needs to be controlled at the micron scale. Under these complex multi-physics coupling conditions, short circuits are prone to occur between the two electrodes, resulting in damage or even scrapping of the anode workpiece and cathode electrode, as well as damage to the power supply. Therefore, a pulse power supply requires a precise and responsive short circuit protection system to promptly disconnect power in the event of a short circuit, thereby reducing machining losses.

**Figure 14:**
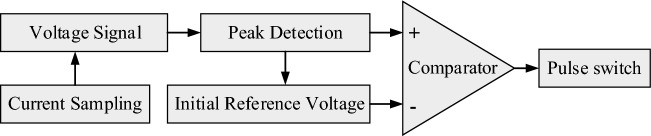
Core schematic diagram of the power protection system.

**Figure 15:**
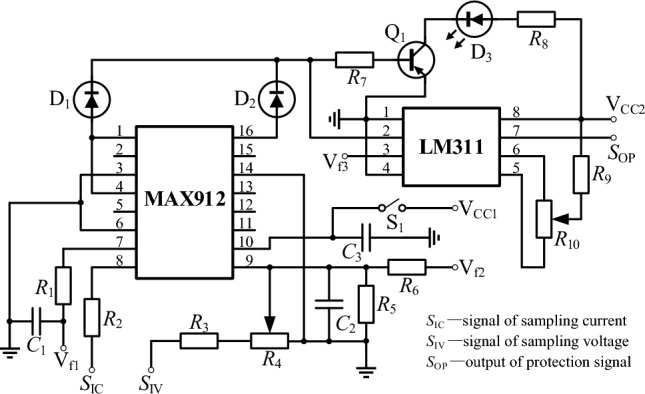
Fast protection circuit.

The power protection system consists of two main components: short-circuit detection and a fast-cutting device, as illustrated in the core schematic diagram in Fig. 14. The short-circuit detection circuit employs a Hall sensor (with a range of 0–5 mA) to monitor the machining current and convert it into an equivalent voltage signal within a certain range (0–10 V). The output equivalent voltage signal is then promptly detected by a peak detection circuit for changes in the peak current of the machining circuit. By comparing the result with the initial reference voltage using a comparator, the circuit can accurately determine whether a short circuit has occurred during the machining process. When the current or voltage exceeds the set reference value, the comparator immediately outputs a high-level protection signal, which is used to cut off the pulse generation and power amplification circuits. The fast protection circuit of the system is depicted in Fig. 15.

## Prototype verification of spike power supply

### Power parameter test

**Figure 16:**
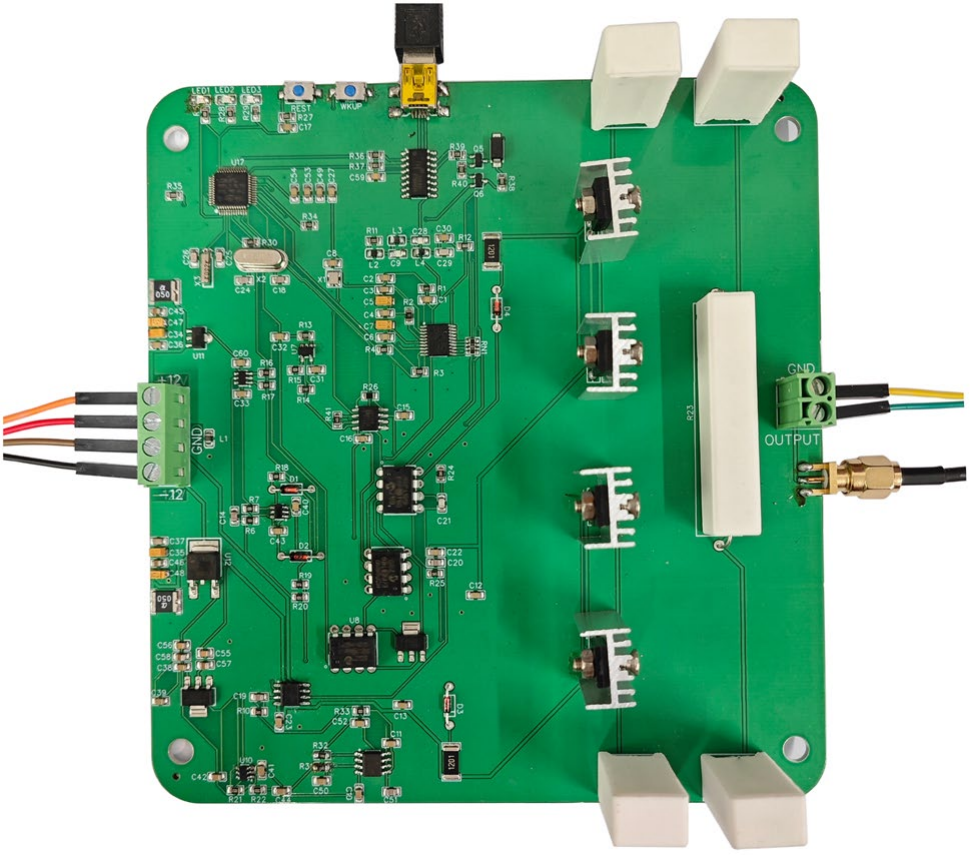
Power supply experimental prototype.

The main experimental prototype of the spike pulse power supply is depicted in Fig. 16. Through actual electrolytic machining tests, the parameters of the spike pulse power supply experimental prototype are shown in Table 1. The maximum pulse frequency of the spike power supply is 20 MHz, and the minimum pulse width is approximately 1.8 ns (*τ* = 2.6 ns).

**Table 1:** Parameter of spike pulse power supply experimental prototype.

Parameter	Value
Voltage amplitude range (V)	0–10
Processing current range (A)	0–1
Maximum pulse frequency (MHz)	20
Minimum pulse width (ns)	1.8
Maximum output power (W)	10
Protection circuit response time (μs)	≤ 50

**Figure 17:**
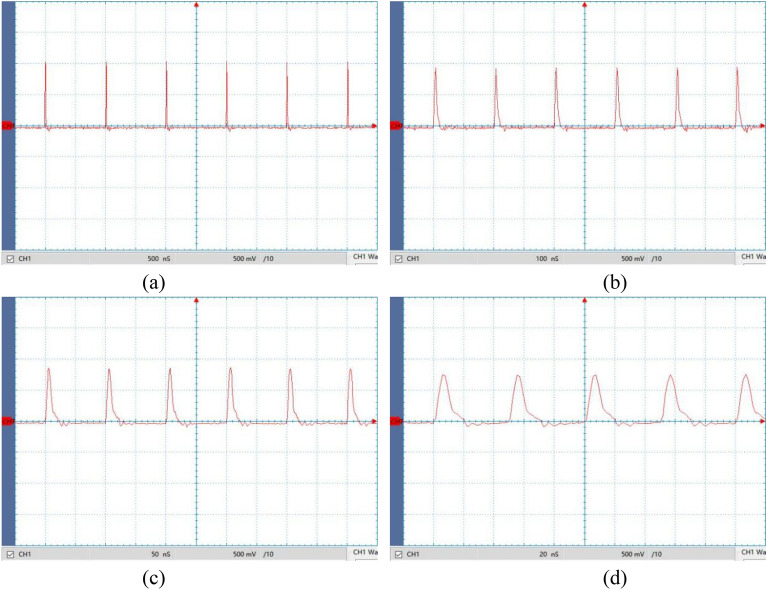
Spike voltage waveforms at different frequencies. (**a**) *f* = 1 MHz; (**b**) *f* = 5 MHz; (**c**) *f* = 10 MHz; (**d**) *f* = 20 MHz.

For the power supply experimental prototype being machined, a fixed-value precision ceramic capacitor with a capacitance of 0.1 nF is used in the differential circuit. The resistance *R* employs an adjustable precision potentiometer, with an adjustment range of 0.2 ~ 200 Ω. By adjusting the differential circuit resistance to set the time constant *τ* = 3.43 ns, the spike pulse frequency is set to 1 MHz, 5 MHz, 10 MHz, and 20 MHz respectively using a microcontroller, with a rectangular pulse peak voltage of *U*_Pm_ = 2 V and a duty cycle of 50%. The spike pulse output waveform is collected using a high-speed oscilloscope, as shown in Fig. 17.

Figure 17 shows that, for a constant time constant, the peak voltage of the spike pulse decreases as the frequency increases. Specifically, at frequencies of 1 MHz, 5 MHz, 10 MHz, and 20 MHz, the peak voltages are *U*_Sm_ = 1.02 V, 0.92 V, 0.85 V, and 0.73 V, respectively. The peak voltage *U*_Sm_ can be adjusted by changing the peak voltage of the rectangular pulse and power amplification gain coefficient. Within the experimental frequency range of 1–20 MHz, the waveform of the spike pulse is steep and stable, and is in good agreement with the theoretical analysis.

**Figure 18:**
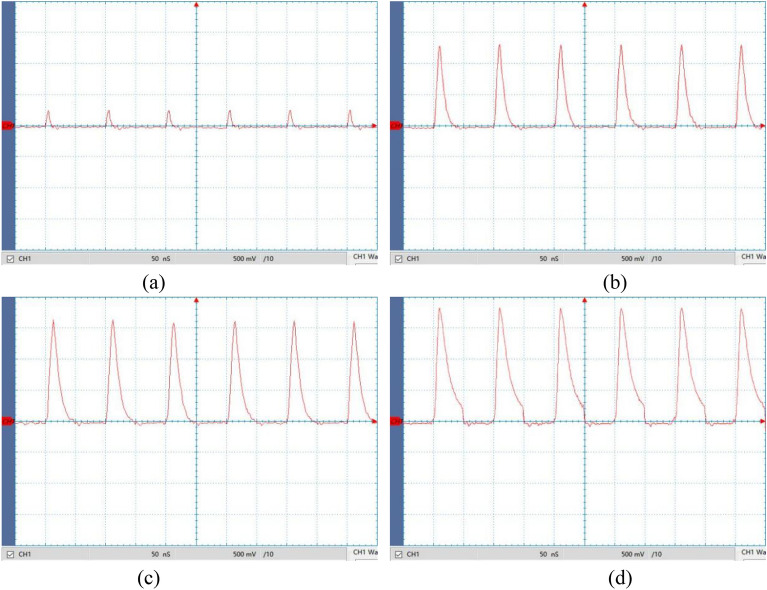
Spike voltage waveforms under different time constants. (**a**) *τ* = 1 ns; (**b**) *τ* = 5 ns; (**c**) *τ* = 10 ns; (**d**) *τ* = 20 ns.

To further investigate the impact of time constants on spike pulse waveforms, we utilized a microcontroller to generate a spike pulse signal with a frequency of 10 MHz, a rectangular pulse peak voltage of *U*_Pm_ = 2 V (2 V for high level, 0 V for low level), and a duty cycle of 50%. The differential circuit's time constants *τ* were adjusted to 1 ns, 5 ns, 10 ns, and 20 ns, respectively. We captured the spike pulse output waveforms using a high-speed oscilloscope, as depicted in Fig. 18.

Figure 18 shows that both the pulse width *S*_w_ and peak voltage *U*_Sm_ of the spike pulse voltage signal increase as the time constant *τ* increases. When *τ* = 1 ns, the pulse width *S*_w_ is 2.4 ns, and the peak voltage *U*_Sm_ is 0.25 V. Although a smaller pulse width can be achieved, the peak voltage is significantly reduced, as shown in Fig. 18a. As the time constant increases, the peak voltage increases sharply (*τ* = 5 ns, *U*_Sm_ = 1.29 V). When *τ* > 5 ns, the rate of change of peak voltage starts to slow down (*τ* = 10 ns, *U*_Sm_ = 1.61 V; *τ* = 20 ns, *U*_Sm_ = 1.82 V). Furthermore, when *τ* > 10 ns, the discharge speed of the differential circuit slows down. During the rectangular pulse duration *t*_on_, the capacitor does not discharge sufficiently and enters the pulse interval stage, resulting in waveform distortion of the spike pulse, as shown in Fig. 18d. When adjusting the spike pulse width through time constant in practical processing applications, it is crucial to ensure that the time constant falls within the allowable duration of the rectangular pulse to prevent waveform distortion.

### ECMM experiment with spike pulse

To evaluate the machining capabilities of spike pulse power, micro-hole electrochemical machining experiments were conducted. The cathode electrode was a tungsten wire with a diameter of 50 μm, while the anode workpiece was a nickel sheet with a thickness of 5 μm. The electrolyte used was a concentrated 0.03 mol/L H_2_SO_4_ solution, and the workpiece feed rate was set at 0.02 μm/s. The parameters of the spike pulse voltage signal are provided in Table 2.

**Table 2:** Setting parameters of spike voltage.

Frequency	Time constant	Pulse width	Peak voltage	
			Case one	Case two
10 MHz	3.43 ns	2.6 ns	1.15 V	1.3 V

**Figure 19:**
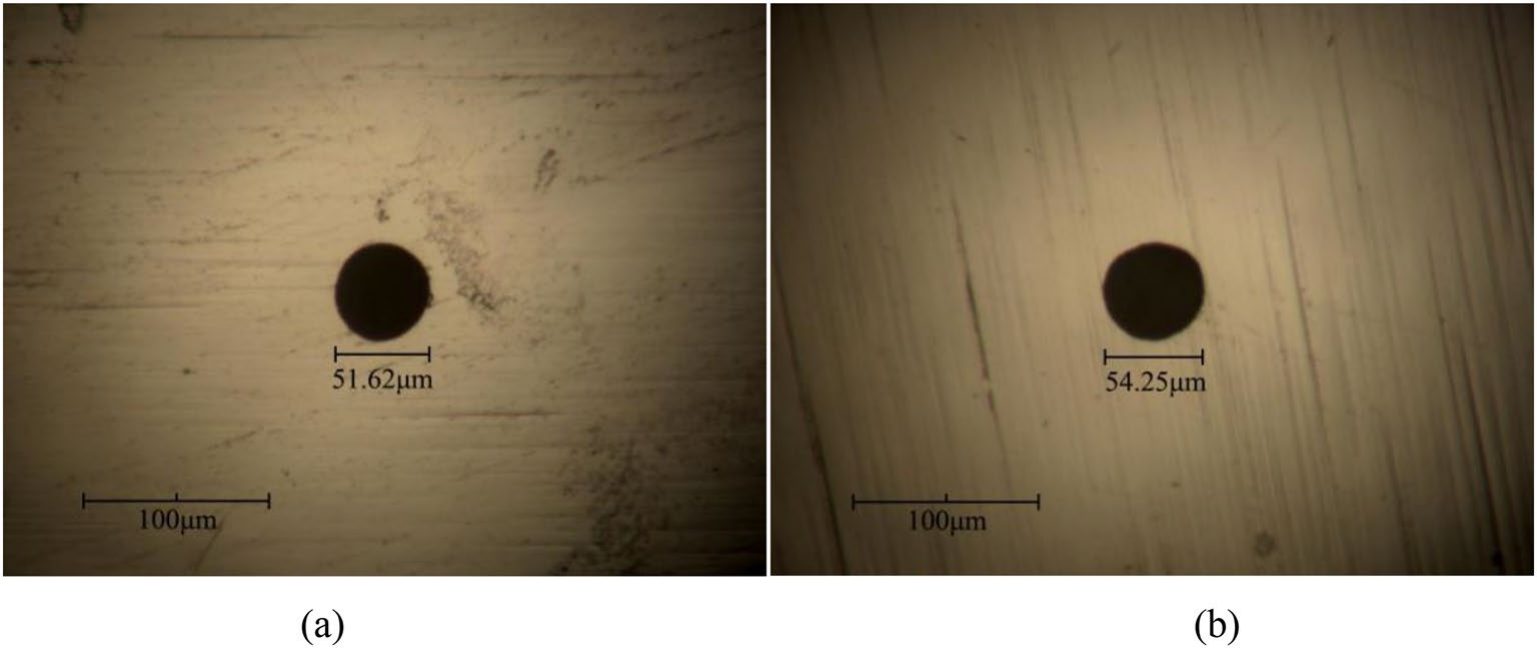
Microhole machined using spike voltage. (**a**) *U*_Sm_ = 1.15 V; (**b**) *U*_Sm_ = 1.3 V.

Figure 19 depicts the experimental results of micro-hole electrochemical machining. In Fig. 19a, micro-holes were machined with a diameter of 51.62 μm and a machining gap of approximately 0.81 μm using a spike pulse peak voltage of 1.15 V (The machining gap reflects the accuracy and localization of electrochemical machining and is calculated as (micro-hole diameter − electrode diameter)/2). in Fig. 19b, micro-holes with a diameter of 54.25 μm and a machining gap of approximately 2.13 μm were machined using a spike pulse peak voltage of 1.13 V. The micro-holes machined with spike pulse voltage exhibited clear edge profiles and no obvious scattered corrosion phenomenon, demonstrating high machining accuracy. These results indicate that the designed spike pulse power source, coupled with low concentration and micro-feed rate process methods, can achieve sub-micron accuracy in electrochemical micromachining.

Furthermore, the validity of the spike pulsed electrochemical micromachining technology has been thoroughly confirmed in both theoretical and experimental aspects in previous investigations^19–21^. Employing this spike pulsed power source, diverse intricate microstructures, such as micro-cantilevers and micro-spiral beams, have been successfully fabricated, enabling the attainment of nanoscale machining clearances.

## Conclusions

This paper presents a high-frequency and narrow-pulse nanosecond spike pulse power supply for ECMM based on a differential circuit design. The design strategy and performance of the power supply are summarized as follows:

1. The main circuit design of the sharp pulse power supply is completed using a microcontroller as the core control element, and stable high-frequency narrow pulse output is achieved through DDS modules and peripheral circuits.
2. The mathematical model of the spike pulse voltage signal is established based on the characteristic analysis of the differential circuit, and the signal waveform is optimized using multiplication and division methods. When the time constant *τ* = 3.43 ns, the optimal parameters for the spike pulse voltage are obtained, with a peak voltage of 1.33 V and a pulse width of 0.81 ns.
3. A prototype of the spike pulse power supply is manufactured and its performance is tested. The maximum pulse frequency of the spike power supply is 20 MHz, and the minimum pulse width is approximately 1.8 ns. This power supply was used in the micro hole electrochemical micromachining experiment and nanoscale machining gaps were obtained.

In addition, the rising/falling time of the rectangular pulse signal used in the theoretical analysis in Sect. 3.3 is the minimum rising/falling time (3.5 ns) of the output signal of the DDS module. However, due to the influence of stray capacitance and inductance in the circuit, the actual rising/falling time of the power supply output signal is about 5 ns, which causes certain errors between the experimental test data and the theoretical analysis results. Therefore, in the subsequent work, we need to further optimize the topology of the power supply circuit and improve the accuracy of the spike pulse voltage waveform.

### Supplementary Information

Supplementary Information.

## Data Availability

All identifed data are available upon reasonable request from the corresponding author.
