# Supplementary material for: Development of nanosecond spike pulse power supply for electrochemical micromachining
Source: Sci Rep. 2023 Dec 21;13:22833. doi: 10.1038/s41598-023-48793-z (PMC10739692; doi:10.1038/s41598-023-48793-z)
Supplement: Supplementary file 1 — Supplementary Information. [file 41598_2023_48793_MOESM1_ESM.docx]

# 2.2 Differential Circuit Design

The differential circuit is the core component that converts rectangular pulse signals into spike pulse waveforms. A differential circuit can significantly reduce pulse width because it only generates an output at the moment when the input waveform experiences a sudden change, while no output is produced during the steady state. The width of the spike pulse waveform output by the differential circuit depends on its time constant *τ* (*τ*=RC). The smaller the time constant, the narrower the spike pulse waveform, and vice versa. To achieve waveform transformation, the time constant of the differential circuit should be smaller than the duration of the rectangular pulse waveform *t*_on_. Generally, a time constant *τ* that is less than or equal to 1/10 of the input waveform duration is selected.

**(1) Differential circuit model**

The principle of waveform transformation in differential circuits is shown in Figure 5. Let *U*_Pm_ be the peak voltage of the input rectangular pulse signal, *t*_rh_ and *t*_rl_ be the rise time and fall time, respectively, *t*_on_ be the pulse duration, and *t*_w_ be the pulse width (the duration of the pulse voltage reaching 90% of the peak voltage), as shown in Figure 5(a). The mathematical expression of the rectangular pulse signal within one period *T* is:

**Fig. 5.** Differential circuit characteristics. (a) Input rectangular pulse waveform; (b) Differential circuit; (c) Output spike waveform.

|  | $U_{\text{P}}=\left\{\begin{matrix} \text{ }\frac{U_{\text{Pm}}t}{t_{\text{rh}}}\text{, 0<}t\leq t_{\text{rh}} \\ \text{ }U_{\text{Pm}},\text{ }t_{\text{rh}}\text{<}t\leq t_{\text{on}}-t_{\text{rl}} \\ -\frac{U_{\text{Pm}}t}{t_{\text{rl}}}+\frac{U_{\text{Pm}}t_{\text{on}}}{t_{\text{rl}}}\text{, }t_{\text{on}}-t_{\text{rl}}\text{<}t\leq t_{\text{on}} \\ \text{ }0,\text{ }t_{\text{on}}\text{<}t\leq T \end{matrix}\right.$ |  |
| --- | --- | --- |

Perform Laplace transformation on Eq. (1) to obtain:

|  | $U_{\text{P}}(s)=\left\{\begin{matrix} \text{ }\frac{U_{\text{Pm}}}{t_{\text{rh}}s^{2}}\text{, 0<}t\leq t_{\text{rh}} \\ \text{ }\frac{U_{\text{Pm}}}{s},\text{ }t_{\text{rh}}\text{<}t\leq t_{\text{on}}-t_{\text{rl}} \\ -\frac{U_{\text{Pm}}}{t_{\text{rl}}s^{2}}+\frac{U_{\text{Pm}}t_{\text{on}}}{t_{\text{rl}}s}\text{, }t_{\text{on}}-t_{\text{rl}}\text{<}t\leq t_{\text{on}} \\ \text{ }0,\text{ }t_{\text{on}}\text{<}t\leq T \end{matrix}\right.$ |  |
| --- | --- | --- |

The differential equation of the differential circuit shown in Fig. 2(b) is:

|  | $U_{\text{O}}+RC\frac{dU_{\text{O}}}{dt}=RC\frac{dU_{\text{I}}}{dt}$ |  |
| --- | --- | --- |

Perform Laplace transform on Eq. (3) to obtain the transfer function of the differential circuit as:

|  | $G(s)=\frac{U_{\text{O}}(s)}{U_{\text{I}}(s)}=\frac{s}{s+1/\tau}$ |  |
| --- | --- | --- |

By combining Eqs. (2) and (4), the Laplace transform of the step response for the differential circuit can be expressed as:

|  | $U_{\text{S}}(s)=G(s)U_{\text{p}}(s)=\left\{\begin{matrix} \frac{U_{\text{Pm}}}{t_{\text{rh}}}\frac{1}{s\left(s+1/\tau\right)}\text{, 0<}t\leq t_{\text{rh}} \\ \frac{U_{\text{Pm}}}{s+1/\tau}\text{, }t_{\text{rh}}\text{<}t\leq t_{\text{on}}-t_{\text{rl}} \\ -\frac{U_{\text{Pm}}}{t_{\text{rl}}}\frac{1}{s\left(s+1/\tau\right)}+\frac{U_{\text{Pm}}t_{\text{on}}}{t_{\text{rl}}\left(s+1/\tau\right)}\text{, }t_{\text{on}}-t_{\text{rl}}\text{<}t\leq t_{\text{on}} \\ 0,\text{ }t_{\text{on}}\text{<}t\leq T \end{matrix}\right.$ |  |
| --- | --- | --- |

To examine the transient response characteristics of the differential circuit, the Laplace inverse transform of Eq. (5) can be performed to obtain the step output response of the differential circuit, which is shown as:

|  | $U_{\text{S}}(t)=\left\{\begin{matrix} \frac{U_{\text{Pm}}\tau}{t_{\text{rh}}}\left(1-e^{-\frac{t}{\tau}}\right)\text{, 0<}t\leq t_{\text{rh}} \\ U_{\text{Sm2}}e^{-\frac{t-t_{\text{rh}}}{\tau}}\text{, }t_{\text{rh}}\text{<}t\leq t_{\text{on}}-t_{\text{rl}} \\ -\frac{U_{\text{Pm}}}{t_{\text{rl}}}\left(1-e^{-\frac{t\text{-}t_{\text{on}}+t_{\text{rl}}}{\tau}}\right)\text{+}U_{\text{Sm3}}\text{, }t_{\text{on}}-t_{\text{rl}}\text{<}t\leq t_{\text{on}} \\ U_{\text{Sm4}}e^{-\frac{t}{\tau}},\text{ }t_{\text{on}}\text{<}t\leq T \end{matrix}\right.$ |  |
| --- | --- | --- |

Considering the issue of voltage drop in circuits, the peak voltage response of a differential circuit *U*_Sm_ will be lower than the peak voltage of the input pulse waveform *U*_Pm_, i.e. *U*_Sm_<U_Pm_ (as shown in Fig. 5(c)). When *t*=*t*_rh_, *t*=*t*_on_-*t*_rl_, and *t*=*t*_on_, the spike pulse output voltage reaches extreme values of *U*_Sm2_, *U*_Sm3_, and *U*_Sm4_, respectively, which are calculated by Eq. (6) as follows:

|  | $\left\{\begin{matrix} U_{\text{Sm2}}=\frac{U_{\text{Pm}}\tau}{t_{\text{rh}}}\left(1-e^{-\frac{t_{\text{rh}}}{\tau}}\right) \\ U_{\text{Sm3}}=U_{\text{Sm2}}e^{-\frac{t_{\text{on}}-t_{\text{rl}}-t_{\text{rh}}}{\tau}} \\ U_{\text{Sm4}}=-\frac{U_{\text{Pm}}}{t_{\text{rl}}}\left(1-e^{-\frac{t_{\text{rl}}}{\tau}}\right)\text{+}U_{\text{Sm3}} \end{matrix}\right.$ |  |
| --- | --- | --- |

The voltage extremes of the spike pulse during each stage are shown in Figure 6. *U*_Sm2_ is the maximum voltage that the spike pulse can reach during the rising stage (*t*≤*t*_rh_) of the rectangular pulse. *U*_Sm3_ is the minimum value of the voltage drop of the spike pulse during the steady-state stage (*t*_rh_<*t*≤*t*_on_-*t*_rl_) of the rectangular pulse. *U*_Sm4_ is the minimum value of the reverse voltage output of the spike pulse during the falling stage (*t*_on_-*t*_rl_<*t*≤*t*_on_) of the rectangular pulse.

**Fig. 6.** Extreme voltage of output spike pulse.

The duration of the spike pulse exceeding 90% of the peak voltage, defined as the pulse width *S*_w_, is shown in Fig. 2(c). Solving Eq. (6), the pulse width of the spike pulse can be obtained as:

|  | $S_{\text{w}}=\tau\ln(0.1+0.9e^{-\frac{t_{\text{rh}}}{\tau}})-\tau\ln(0.9)+t_{\text{rh}}$ |  |
| --- | --- | --- |

The main design parameters of the spike voltage signal include frequency, amplitude, and pulse width. The frequency of the spike voltage signal depends on the frequency of the rectangular wave input to the differential circuit and can be adjusted by the pulse generator circuit. Eq. (7) shows that the peak voltage of the spike pulse *U*_Sm2_, is related to the rise time of the input rectangular pulse *t*_rh_, the peak voltage of the input pulse *U*_Pm_, and the time constant of the differential circuit *τ*. The rise time of the rectangular pulse *t*_rh_ is determined by the waveform characteristics of the input rectangular pulse and cannot be changed, while the peak voltage of the rectangular pulse *U*_Pm_ can be adjusted within a certain range. Therefore, the adjustment of the peak voltage of the spike pulse *U*_Sm2_ can be achieved by adjusting two parameters: the peak voltage of the input rectangular pulse *U*_Pm_ and the gain coefficient of the power amplifier circuit. Eq. (8) shows that the pulse width of the spike pulse *S*_w_ is only related to the rise time *t*_rh_ of the rectangular pulse, and the time constant *τ*. Since it is difficult to adjust the rise time of the rectangular pulse *t*_rh_, the time constant of the differential circuit *τ* is the key parameter that needs to be optimized in the design process of the spike pulse power supply topology.

**(2) Time constant optimization**

The response speed of a differential circuit is determined by the time constant *τ*. When the rise time of the input pulse is fixed, the peak voltage and pulse width of the output spike depend on the time constant. To further optimize the spike waveform, it is necessary to determine the relationship between the time constant of the differential circuit and the output characteristics of the spike pulse.

Assuming a rectangular pulse with a peak voltage of *U*_Pm_=2V, rise/fall times of *t*_rh_=*t*_rl_=3.5ns, period *T*=100ns, and pulse duration *t*_on_=50ns, the waveform of *U*_P_ is obtained using eq. (1) as shown in Fig. 6. Based on the output response function of the differential circuit (eqs. (6) and (7)), we take differential circuit time constants of *τ*=2, 5, 10, and 20ns to investigate the output response of the spike pulse power supply, as shown in Fig. 7. Since there is a reverse output response in the differential circuit during the intermittent stage of the rectangular pulse, the duty cycle of the input rectangular pulse signal is set to 50%.

**Fig. 7.** Output response characteristics of differential circuits. *U*_P_ is the input rectangular pulse voltage waveform

Figure 7 shows that as the time constant *τ* decreases, the peak voltage response of the spike pulse *U*_Sm_ decreases and the spike pulse width becomes narrower. When the time constant *τ*=20ns, the capacitor has not fully charged and enters the pulse-off phase. To complete the spike pulse waveform transformation properly, the time constant *τ* should be at least less than the duration of the input rectangular pulse *t*_on_, i.e., *τ*≤*t*_on_. According to eqs. (7) and (8), the time constant *τ* determines the pulse width *S*_w_ and peak voltage *U*_Sm_ of the spike pulse. Let the peak voltage of the input rectangular pulse *U*_Pm_=2V and the duration of the pulse *t*_on_=50ns, with a rise time *t*_rh_=3.5ns. Using Matlab, the variation of *S*_w_ and *U*_Sm_ with *τ* can be obtained as shown in Figure 8.

**Fig. 8.** The variation of spike pulse width *S*_on_ and peak voltage *U*_Sm_ with time constant *τ.*

Figure 8 shows that as the time constant increases, the spike pulse width *S*_w_ first decreases sharply and then gradually increases. When the time constant *τ*=2.6ns, the spike pulse width *S*_w_ reaches its minimum value of 0.78ns. This is because when the time constant *τ* is small, the response speed of the differentiator circuit is extremely fast, and the capacitor of the differentiator circuit can be charged during the rising stage of the rectangular pulse (*t*≤*t*_rh_). The output waveform of the differentiator circuit reaches a stable state, which is no longer a spike pulse, but rather a rectangular pulse with a small amplitude (as shown in Fig. 9). Therefore, during the 0<*t*<2.6ns stage, the spike pulse width *S*_w_ decreases with an increase in the time constant *τ*. When *τ*>2.6ns, the spike pulse width *S*_w_ increases proportionally with an increase in the time constant *τ*. Based on the above analysis, to ensure the proper transformation of the spike pulse waveform, the time constant *τ* should be set to at least 2.6ns.

**Fig. 9.** The voltage output waveform of the differential circuit when *τ*<2.6ns.

Furthermore, the functional relationship between *U*_Sm_ and *τ* in Fig. 8 indicates that the peak voltage of the spike pulse *U*_Sm_ increases rapidly with the time constant *τ*, but then increases at a slower rate until it gradually approaches the peak voltage of the rectangular pulse *U*_Pm_. A smaller time constant *τ* results in a smaller peak voltage of the spike pulse, which in turn leads to a greater voltage drop generated by the differential circuit. Therefore, by adjusting the value of the time constant *τ*, the peak voltage and pulse duration of the spike pulse can be tuned, providing a theoretical basis for adjusting and optimizing the parameters of the spike pulse power supply.

To achieve higher machining accuracy and localization in PECMM, it is necessary to reduce the material removal per pulse of the anode by minimizing the pulse width of the machining power supply. If the time constant *τ* of the differential circuit is too small, a smaller spike pulse width *S*_w_ can be obtained, but the peak voltage of the spike pulse output *U*_Sm2_ will be very small, resulting in a large voltage drop across the differential circuit. Conversely, when the time constant *τ* is relatively large, the spike peak voltage *U*_Sm2_ will increase and the voltage drop across the circuit will decrease, but the spike pulse width *S*_w_ will also increase and the differential effect will become less obvious. To balance the contradiction between peak voltage *U*_Sm2_ and pulse width *S*_w_, the selection of the time constant *τ* of the differential circuit is optimized by introducing a multiplication and division optimization objective function *Y*. The definition of *Y* is as follows:

|  | $Y=\frac{U_{\text{Sm2}}}{S_{\text{w}}}\text{=}\frac{U_{\text{Pm}}\tau}{\tau t_{\text{rh}}\ln(0.1+0.9e^{-\frac{t_{\text{rh}}}{\tau}})-\tau t_{\text{rh}}\ln(0.9)+t_{\text{rh}}^{2}}\left(1-e^{-\frac{t_{\text{rh}}}{\tau}}\right)$ |  |
| --- | --- | --- |

In eq. (9), the unit of the objective function *Y* is V/s, which can be regarded as the rate of voltage change. The larger the peak voltage *U*_Sm2_ and the smaller the pulse width *S*_w_, the higher the value of the objective function *Y*. When the objective function *Y* reaches its maximum value, it can ensure that the differential circuit obtains a narrow pulse width without excessive voltage drop. Taking *U*_Pm_=2V and *t*_rh_=3.5ns, the relationship between the objective function *Y* and the time constant *τ* can be obtained by solving eq. (9), as shown in Fig. 10.

**Fig. 10.** Functional relationship between objective function Y and time constant *τ*.

Fig. 10 shows that the objective function *Y* is a unimodal curve. The maximum value of *Y*_max_=5.5×10^8^ V/s is obtained when the time constant *τ*=3.43 ns. By substituting *τ*=3.43ns into eqs. (7) and (8), the peak voltage *U*_Sm2_ and the spike pulse width *S*_w_ can be obtained:

|  | $\left\{\begin{matrix} U_{\text{Sm2}}=1.33\text{V} \\ S_{\text{w}}=0.81\text{ns} \end{matrix}\right.$ |  |
| --- | --- | --- |

Through optimization analysis of the objective function, it was found that by setting the time constant of the differential circuit to 3.43 ns, a rectangular pulse signal with a duration of 50 ns and peak voltage of 2V can be converted to a narrow pulse signal with a pulse width of 0.81 ns and peak voltage of 1.33V. By substituting *τ*=3.43 ns into eqs. (6) and (7), the optimized output waveform of the narrow pulse is shown in Fig. 11, where the pulse width is shortened by about 1/62 of the original input signal, and the voltage drop is approximately 0.67V. When the time constant *τ* is further decreased, the pulse width of the narrow pulse does not decrease significantly, but the peak voltage decreases significantly.

After a comprehensive analysis, the optimal range of time constant *τ* for the differential circuit is between 2.6ns and 0.1*t*_on_ (*t*_on_≥26ns) to improve the precision of ECMM and ensure complete transformation of the spike pulse waveform. Specifically, when the pulse width *t*_on_ is 50ns, the range of time constant should be 2.6ns ≤ *τ* ≤ 5ns. When the time constant τ is set to 2.6ns, the maximum allowed input rectangular pulse frequency is *f*_P_=19.23MHz. At this point, the output frequency *f*_S_ of the spike pulse signal after the full-wave rectification circuit is 2*f*_P_=38.46MHz.

**Fig. 11.** Optimized spike pulse waveform. (*τ*=3.43ns; *U*_Pm_=2V; *t*_r_=3ns; *t*_on_=50ns)
